# A small notch width index, steeper medial and lateral tibial slope and higher lateral/medial tibial slope ratio are relevant knee morphological factors for ACL injuries in skeletally immature patients—A systematic review

**DOI:** 10.1002/jeo2.70211

**Published:** 2025-03-22

**Authors:** Robin Voskuilen, Martijn Dietvorst, Marieke van der Steen, Rob P. A. Janssen

**Affiliations:** ^1^ Department of Orthopaedic Surgery & Trauma Máxima Medical Center Eindhoven the Netherlands; ^2^ Department of Orthopaedic Surgery & Trauma Catharina Hospital Eindhoven Eindhoven the Netherlands; ^3^ Orthopaedic Biomechanics, Department of Biomedical Engineering Eindhoven University of Technology Eindhoven the Netherlands; ^4^ Chair Value‑Based Health Care, Department of Paramedical Sciences Fontys University of Applied Sciences Eindhoven the Netherlands

**Keywords:** anterior cruciate ligament, knee morphology, notch width index, paediatric, skeletally immature, tibial slope

## Abstract

**Purpose:**

The incidence of anterior cruciate ligament (ACL) injuries in skeletally immature patients has drastically increased over the last decades. Morphology of the knee might play an important role. This literature review provides a systematic overview of knee morphological factors relevant to ACL injury in skeletally immature patients. The hypothesis of the present study is that multiple knee morphological parameters—such as a steep medial and lateral tibial slope (MTS and LTS) and a narrow intercondylar notch—can be identified as potentially relevant factors for ACL injury in this population.

**Methods:**

Systematic review according to PRISMA guidelines. MEDLINE, Embase and Cochrane were searched in December 2023 for studies reporting on knee morphology and ACL injury in skeletally immatures. The following inclusion criteria were used: English/Dutch studies, full‐text available, human studies and skeletally immature patients. Parameters with clinical homogeneity and presented in two or more studies as means with standard deviation were included in a meta‐analysis using RevMan. Parameters that could not be included in the meta‐analyses were presented in a descriptive manner.

**Results:**

After screening 1825 studies, a total of 18 studies were included, of which 16 studies had parameters included in the meta‐analyses. These studies investigated 31 knee morphological factors for ACL injury in skeletally immatures. Meta‐analyses identified a smaller notch width index (NWI) (0.25 vs. 0.26, mean difference: −0.02 95% confidence interval [CI]: −0.03 to −0.01, *p* ≤ 0.00001) steeper MTS and LTS (4.8° vs. 3.6° (mean difference: 0.55° 95% CI: 0.09–1.01, *p* = 0.02) and 4.3° vs 2.8° (mean difference: 2.04° 95% CI: 0.75–3.32, *p* = 0.0003), respectively) and higher LTS/MTS ratio as risk factors for ACL injury in skeletally immature patients.

**Conclusions:**

A small NWI, steeper MTS and LTS and higher LTS/MTS ratios were identified as relevant knee morphological factors for ACL injuries in skeletally immature patients.

**Level of Evidence:**

Level III.

**Abbreviations**
ACL: anterior cruciate ligament
LMBA: lateral meniscal bone angle
LTS: lateral tibial slope
LTS/MTS ratio: lateral/medial tibial slope ratio
MTS: medial tibial slope
NOS: Newcastle‐Ottawa scale
NWI: notch width index
PRISMA: Preferred Reporting Items for Systematic Reviews and Meta‐Analyses
RIA: roof inclination angle
TE: tibial eminence

## BACKGROUND

Anterior cruciate ligament (ACL) injuries are severe knee injuries with a substantially increased risk of osteoarthritis [53]. The last decades show an increase in ACL injuries in children and adolescents, mostly due to higher sports participation at a younger age [6, 9, 19, 25]. Both intrinsic and extrinsic factors can play a role in ACL injury [47]. Examples of intrinsic factors are gender, hormonal milieu, genetic factors, neuromuscular function and knee morphology [2]. Extrinsic factors can include playing on the surface, sports level and weather [47].

The role of knee morphology in relation to ACL injury and ACL graft rupture has extensively been studied in the last few years. In adults, these risk factors are well described and include intercondylar notch stenosis, variations in sagittal condylar shape, increased tibial slope, reduced tibial eminence size, poor tibiofemoral congruity as well as reduced ACL size as substantial risk factors for ACL injury [5]. An enhanced understanding of knee morphology is factored into treatment decisions. To date, no studies have been performed on adjusting knee morphology to prevent ACL injuries in humans. In ACL revision surgery, knee morphology is taken into account in the treatment decision such as an increased tibial slope and treated if necessary [42].

In children and adolescents, there is no current overview of knee morphological factors for ACL injury. The question remains whether the same anatomical factors play a role in this population as in adults. Differences may be related to the growth and maturation of bones and the interpretation of the diagnostic images [31].

The purpose of the present study is to identify and assess differences in knee morphology between skeletally immature patients with and without primary ACL injuries. The hypothesis of the present study was that multiple knee morphological parameters—such as a steep medial and lateral tibial slope (MTS and LTS) and a narrow intercondylar notch—can be identified as potentially relevant factors for ACL injury in the skeletally immature patient.

## MATERIALS AND METHODS

### Protocol

The study protocol was registered at Prospero (ID: CRD42022370644) on 26 October 2022. This systematic review was conducted and presented according to the PRISMA (Preferred Reporting Items for Systematic Reviews and Meta‐Analyses) guidelines [44].

### Eligibility criteria

Studies that fulfilled the following criteria were included: studies on skeletally immature patients with primary ACL injuries reporting morphological parameters of the knee and were compared with a healthy control group. Only studies published in English or Dutch were considered (Table 1).

**Table 1:** Inclusion and exclusion criteria.

Inclusion	Exclusion
Skeletally immature patients with primary ACL injury	Multi‐ligament or non‐ACL knee injuries and skeletally mature patients
Morphological parameters of the knee (such as tibial slope and notch width)	
Studies involving non‐ACL‐injured skeletally immature controls	Non‐human studies, technique studies and case reports
English and Dutch language studies	No full text available/conference abstracts

Abbreviation: ACL, anterior cruciate ligament.

### Search strategy

One investigator (ED) performed a comprehensive systematic review of the literature using PubMed, EMBASE, and Cochrane from inception to 31 December 2023.

Search terms included MESH terms related to, ‘Anterior cruciate ligament’, ‘Anterior cruciate ligament injuries’, ‘Tibial slope’, ‘Morphometry’, ‘Morphology’ and ‘Paediatric’. Duplicates were removed. In addition, reference lists of included articles were screened for relevant additional references.

### Selection procedure

Two researchers (RV and MD) independently screened titles and abstracts for eligibility using the Rayyan software [17]. A full‐text version of all eligible studies was reviewed on the in‐ and exclusion criteria by two researchers (RV and MD). Any disagreements between the reviewers were resolved by discussion.

### Data extraction

One researcher (RV) extracted all relevant data, and the other researcher (MD) independently checked all extracted data. The data included population characteristics, study methods, morphological outcomes per group and measurement methods (e.g., magnetic resonance imaging [MRI] and radiographs). Demographic data and data pertaining to the morphology of the injured and uninjured populations were extracted from the included studies.

### Risk of bias

Non‐randomized controlled trials (non‐RCTs) were evaluated using the Risk of Bias in Nonrandomised Studies of Interventions (NRSI) (ROBINS‐I) tool [64]. Seven domains of potential bias in NRSI were assessed. Possible confounding and the nature of patient selection before the start of the comparative intervention are assessed by two domains. A further domain is used to assess bias in the classification during the intervention. The final four domains assess the methodological quality after the intervention comparison has been implemented and relate to deviations from previously intended interventions, missing data, erroneous measurement of outcomes and bias in the selection of reported outcomes.

### Data analysis

Parameters with clinical homogeneity and presented in two or more studies as means with standard deviation were included in the meta‐analysis. In studies with missing data, corresponding authors were contacted. Statistical heterogeneity was estimated with the use of a standard chi‐square test and the *I* ^2^ statistics. An *I* ^2^ statistic value of >50% was considered to indicate substantial heterogeneity. If statistical heterogeneity is not substantial (*I* ^2^ ≤ 50% or *p* > 0.05), a fixed effect model will be used. If statistical heterogeneity is substantial (*I* ^2^ ≥ 50% or *p* < 0.05), the random effects model will be used. All mean differences were used for the meta‐analysis. The meta‐analyses were carried out using the RevMan 5 software package (Cochrane Collaboration). Demographic data and data pertaining to the morphology of the injured and uninjured populations were extracted from the included studies. The standard mean differences were reported as an effect measure. Parameters that could not be included in the meta‐analyses are presented descriptively.

## RESULTS

**Figure 1:**
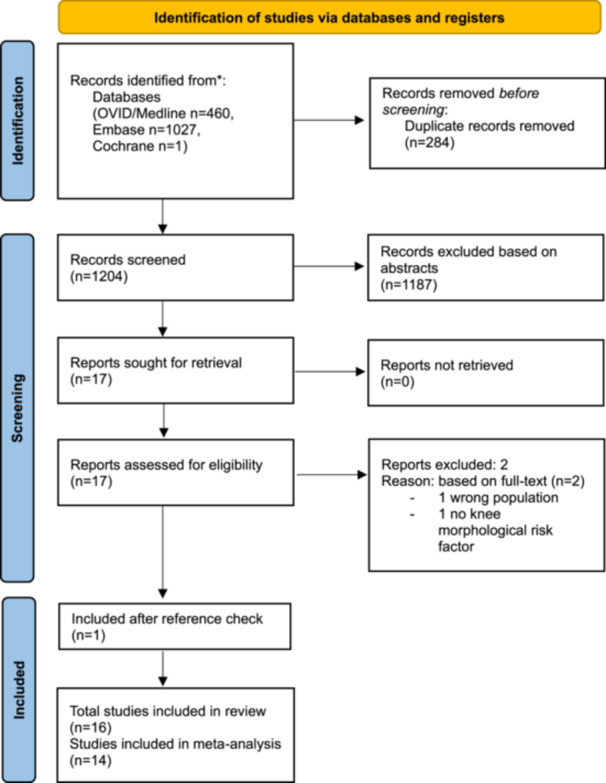
Flowchart inclusion. In total, 1825 hits of which 1456 were screened after removing duplicates. Seventeen studies were included, and one study was added after reference check. Sixteen studies were used for the meta‐analysis, and two studies were used for descriptive results.

The search strategy included 1825 results. After duplicates, non‐full‐text, non‐English and non‐human studies were removed, and 1456 studies proceeded to systematic screening (Figure 1). After screening of titles, abstracts and full texts, 18 studies were included [1, 13, 14, 20, 22, 26, 27, 37, 38, 39, 43, 46, 50, 51, 52, 57, 59, 66]. Two studies [51, 57] did not describe a standard deviation in their results and could not be included in the meta‐analysis. A total of 16 studies were used for the meta‐analysis.

### Risk of bias

**Figure 2:**
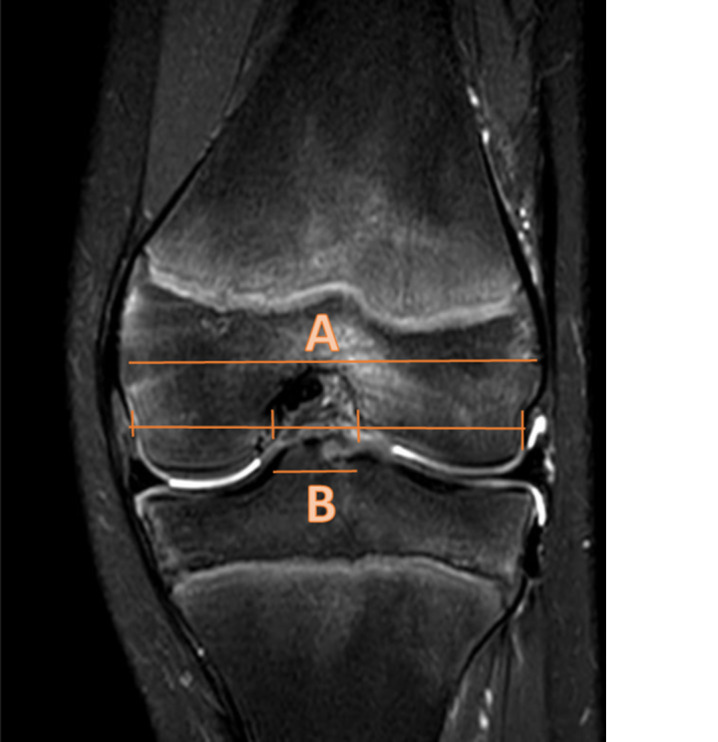
Notch width index (NWI). The NWI is measured on MRI by measuring the bicondylar width at the level of the popliteal groove in the lateral condyle of the femur, parallel to the joint line as formed by the distal femoral condyles (A). Then the most inferior margins of the intercondylar notch are identified. The distance between these points represents the intercondylar notch width (B). The NWI represents the ratio of the intercondylar notch width to the bicondylar width (B/A) [55]. MRI, magnetic resonance imaging.

Sixteen studies were classified as low‐risk bias studies according to Robins‐I tool [1, 13, 14, 20, 22, 26, 27, 37, 38, 39, 50, 51, 52, 57, 59, 66], and two studies were classified as studies with moderate risk bias [43, 46] (Figure 2).

### Study characteristics

The 18 included studies comprised 2247 patients ranging in age from 7.6 to 17.0. In 16 studies, MRI was used for diagnostics, while in 2 studies, knee radiographs were used. A total of 31 knee morphological parameters risk factors were identified as potential risk factors in the 18 studies (Table 2).

**Table 2:** Risk of bias based on the ROBINs‐I tool per study [16].

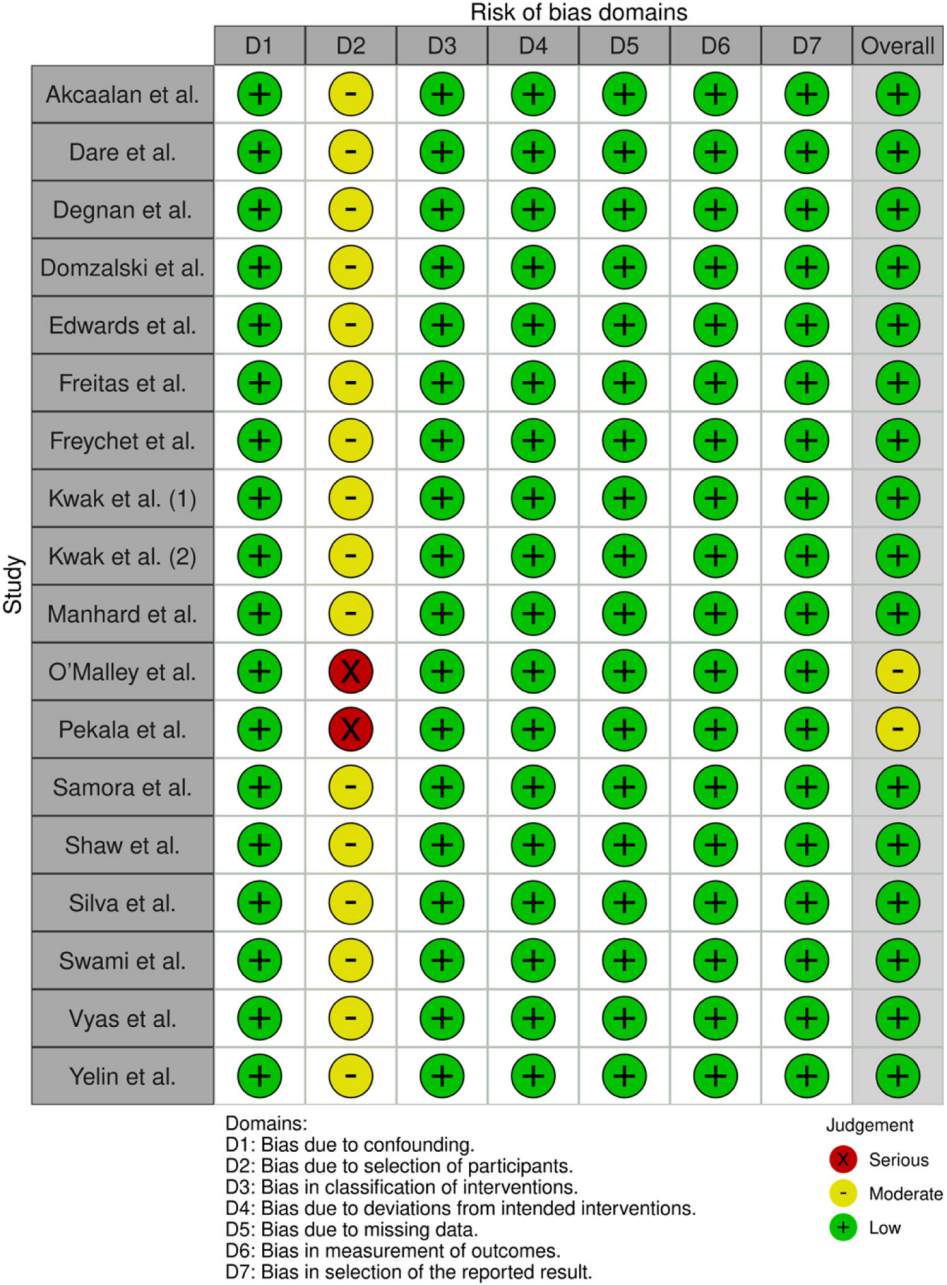

Factors were mainly related to notch (index, width and volume) [1, 20, 26, 27, 39, 46, 50, 51, 52, 57, 59, 66], tibial slope (medial/lateral/ratio/depth/posterior) [13, 22, 26, 38, 43, 50, 57, 59] and patellar height [14, 37]. In addition, femoral‐related factors (e.g., intercondylar spine [27], femoral trochlea dysplasia [37] and anatomic differences in the femur [38]), as well as tibial‐related factors (e.g., meniscal bone angle [22], meniscal slope [26], roof inclination [50] and tibial eminence [51]) were assessed. Considering clinical homogeneity related to imaging technique and method to measure the morphological parameter, meta‐analyses could be performed for six parameters. The remaining parameters could only be included in a descriptive manner (Table 3).

**Table 3:** Characteristics of the included studies.

					Number of patients		Age		% Male		% Right side	
Author, year	Study design	Image technique	Morphological parameter(s)	Trauma mechanism	Controls	Cases	Controls	Cases	Controls	Cases	Controls	Cases
Akcaalan et al. 2023 [1]	Case–control study	MRI	MTS, NWI, Medial plateau depth	NR	115	115	10.44 (10–17)	10.96 (10–17)	53	66	51	53
Dare et al. 2015 [13]	Case–control study	MRI	LTS, MTS, LTS/MTS ratio	Non‐contact trauma	76	76	14.8 ± 1.3	14.8 ± 1.3	46	46	NR	NR
Degnan et al. 2015 [14]	Case–control study	MRI	Insall–Salvati ratio	NR	34	36	12.8 ± 2.1	12.4 ± 1.4	81	74	83	68
Domzalski et al. 2010 [20]	Retrospective case–control study	MRI	NWI	Known in 42 knees (91%) – Non‐contact trauma 34 knees (74%) – Contact trauma 12 knees (26%)	46	44	14.5 (11.4–16.9)	14.7 (11.5–16.9)	43	41	43	54
Edwards et al. 2021 [22]	Case–control study	MRI	LTS, MTS, slope difference, LTS/MTS ratio, Lateral MBA, LTS/LMBA ratio	Non‐contact (100%)	20	20	14.80 ± 2.42	14.80 ± 2.42	75	75	NR	NR
Freitas et al. 2021 [26]	Retrospective case–control study	MRI	NWI, LTS, MTS, MMS, LMS	NR	140	433	NR	NR	47	75	NR	NR
Freychet et al. 2016 [27]	Case–control study	MRI	NWI, Tibial slope, alpha angle, intercondylar spine	NR	49	50	13.8 (11.7–15.9)	13.6 (12.1–15.1)	32	67	50	49
Kwak et al. 2016 [37]	Case–control study	MRI	Insall–Salvati, femoral dysplasia (Dejour)	Non‐contact trauma	116	115	14.2 ± 3.6	14 ± 3.7	75	74	NR	NR
Kwak et al. 2016 [38]	Case–control study	MRI	LTS, MTS, anteroposterior length, sagittal curvature	Non‐contact trauma	100	100	14.4 ± 3.6	14.5 ± 3.8	75	73	NR	NR
Manhard et al. 2023 [39]	Case–control study	MRI	TC, TSH, MFC, NWI, NHI	NR	72	49	7.6 ± 2.2 (4.0–11.0)	10.1 ± 1.5 (5.0–11.9)	47	55	NR	NR
O'Malley et al. 2017 [43]	Retrospective case–control study	X‐ray	Posterior tibial slope	NR	32	32	13.1 (9–16)	13.1 (9–17)	75	72	41	38
Pekala et al. 2019 [46]	Retrospective case–control study	MRI	NWI	NR	35	39	NR	NR	38	34	NR	NR
Samora et al. 2016 [50]	Case–control study	X‐ray	Posterior tibial slope, NWI, roof inclination angle	NR	25	50	NR	NR	NR	NR	NR	NR
Shaw et al. 2015^a^ [51]	Retrospective case–control study	MRI	Notch width, NWI, notch volume, LTS, MTS, LTS depth, MTS depth, TE height, TE volume, TE width	NR	28	39	14.29 ± 1.0	14.25 ± 2.0	50	54	NR	NR
Silva et al. 2021 [52]	Case–control study	MRI	NWI	NR	11	11	11.36 ± 1.9	12.8 ± 1.2	91	73	73	27
Swami et al. 2013^a^ [57]	Retrospective case–control study	MRI	NWI, Notch width, Notch volume	NR	50	50	14.2 ± 0.7	14.8 ± 0.9	56	56	NR	NR
Vyas et al. 2010 [59]	Retrospective case–control study	X‐ray	MTS, LTS, NWI	NR	16	23	14.4	16.3	NR	NR	NR	NR
Yelin et al. 2021 [66]	Retrospective case–control study	MRI	NWI	NR	22	23	14.48	14.52	NR	NR	NR	NR

Abbreviations: LMBA, lateral meniscal bone angle; LMS, lateral meniscal angle; LTS, lateral tibial slope; MFC, medial femoral condyle; MMS, medial meniscal angle; MTS, medial tibial slope; NHI, notch height index; NWI, notch width index; NR, not reported; TC, transcondylar; TE, tibial eminence; TSH, tibial spine height.

^a^ Studies were not included in the meta‐analysis because results could not be used for the meta‐analysis.

### Notch

#### Notch width index (NWI)

**Figure 3:**
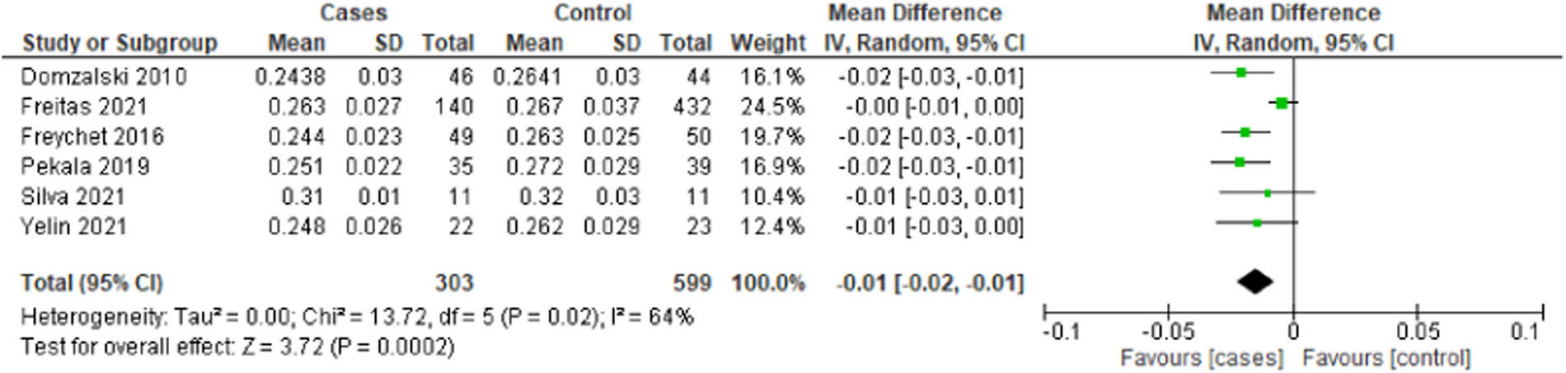
Meta‐analysis from studies evaluating the notch width index. CI, confidence interval; SD, standard deviation.

Twelve studies measured the NWI (Figure 3), of which 2 studies measured the NWI on a knee radiograph [50, 59] and 10 studies measured NWI on MRI [1, 20, 26, 27, 39, 46, 51, 52, 57, 66]. Of these 10 MRI studies, 8 studies were included in the meta‐analysis [1, 20, 26, 27, 39, 52, 66].

**Figure 4:**
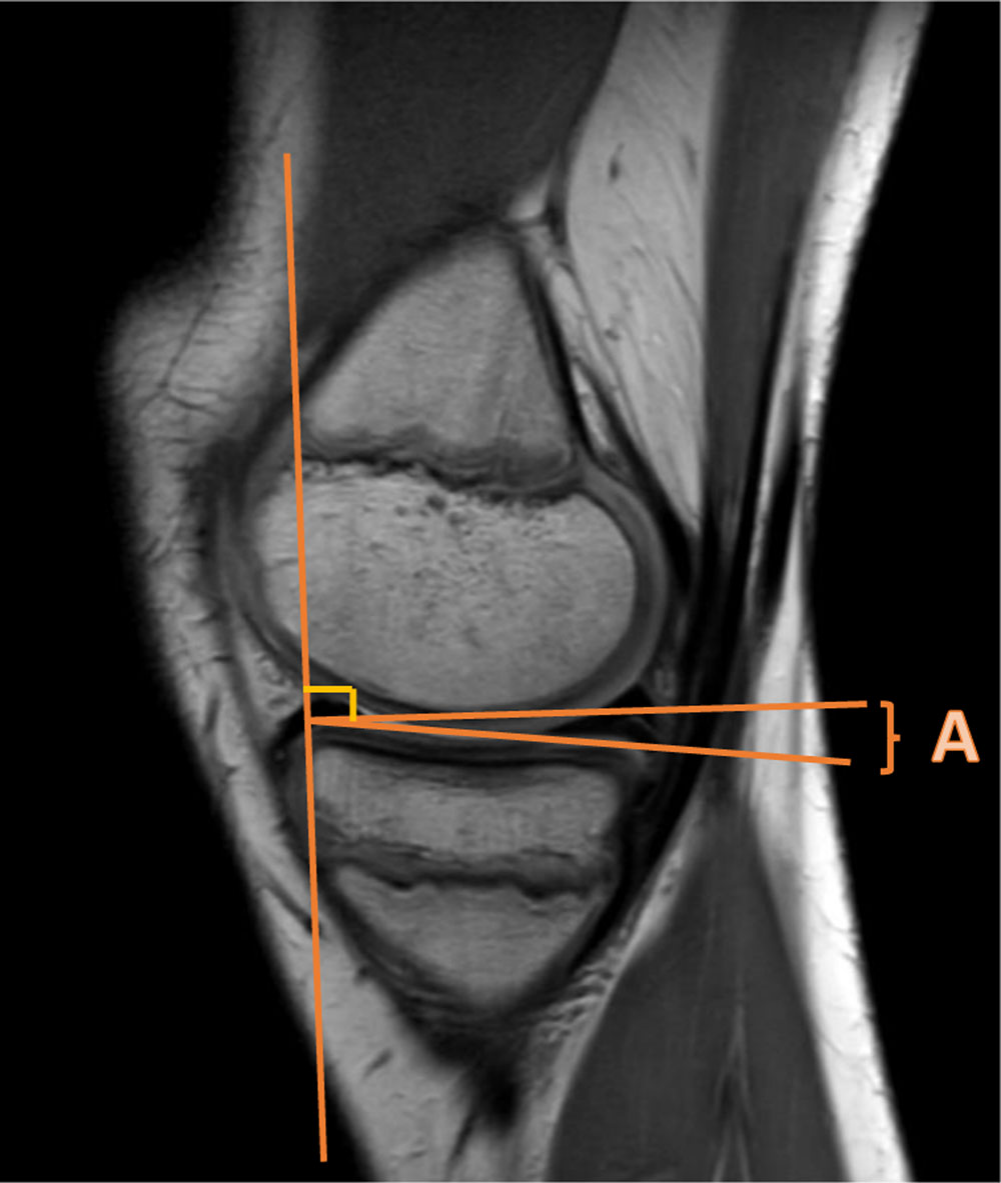
Medial tibial slope. Medial tibial slope was measured by Hudek's technique [33].

Meta‐analysis showed that the average NWI of the ACL‐injured group was significantly smaller than in the control group with a mean difference of −0.02 (95% confidence interval [CI]: −0.03 to −0.01, *p* ≤ 0.00001), random effect model. The average NWI of the ACL‐injured group was 0.25 in the ACL‐injured group versus 0.26 in the control group (Figure 4). The two studies measuring the NWI on a knee radiograph did not find significant differences in NWI between the ACL‐injured group and the control group [50, 59].

#### Notch volume

The difference in notch volume was assessed in two studies [51, 57]. Swami et al. [57] found a significantly smaller notch volume in ACL‐injured patients (*p* = 0.002). Shaw et al. [51] did not find a difference between the control group and the ACL‐injured group.

#### Notch width

Shaw et al. [51] and Swami et al. [57] assessed the notch width, which could not be included in the meta‐analysis. Both studies did not find a difference between the ACL‐injured group and the control group.

#### Notch roof height and notch height index

Akcaalan et al. [1] assessed the notch roof height and notch height index. The notch height index was assessed by dividing the notch roof height by the medial femoral condyle height. The notch roof height was significantly (*p* = 0.028) higher in ACL‐injured patients compared to the control group. No significant (*p* = 0.051) difference was found in the notch height index between the ACL‐injured group and the control group.

### Tibial slope

**Figure 5:**
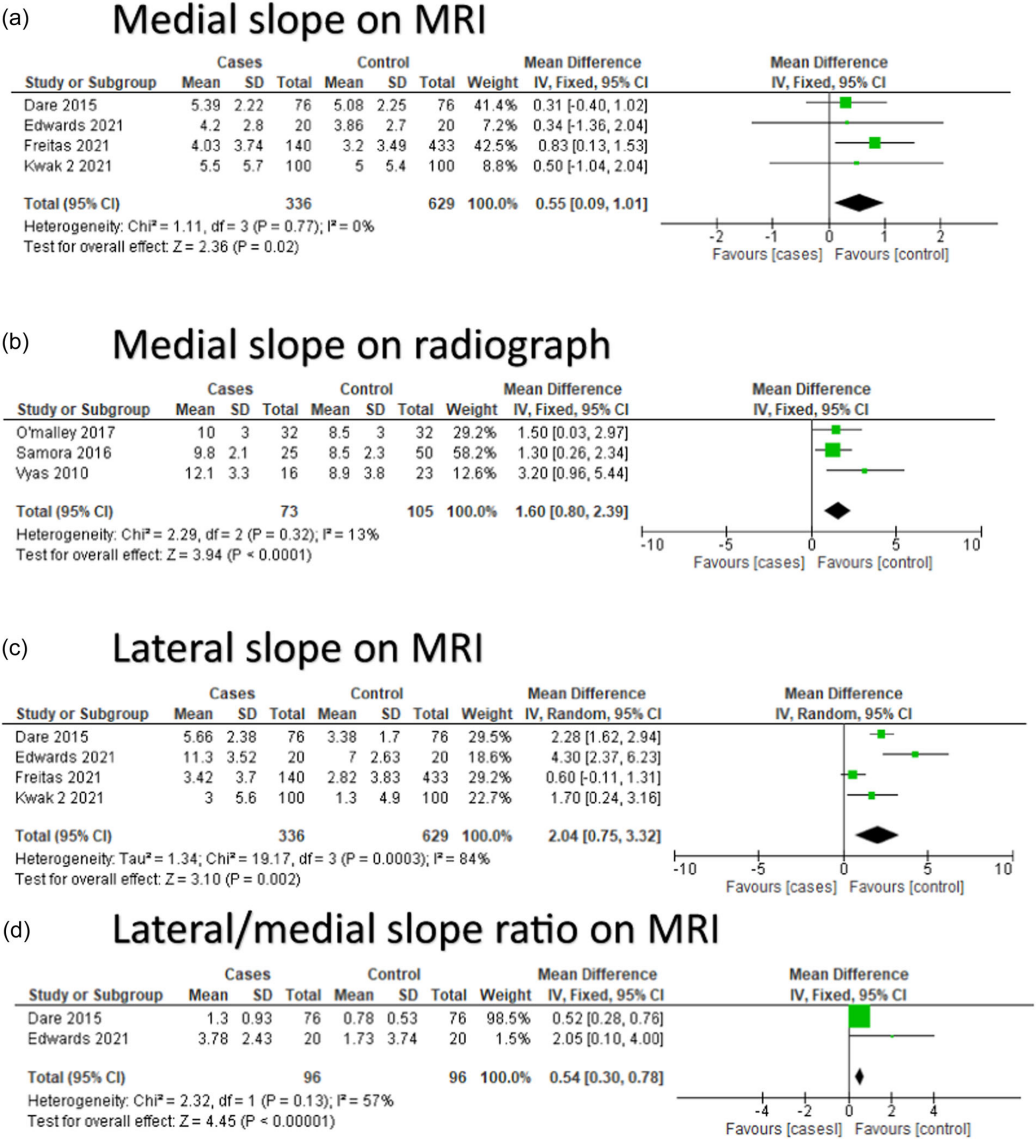
Meta‐analyses of tibial slope. (a) Medial slope on MRI. (b) Medial slope on x‐ray. (c) Lateral slope on MRI. (d) Lateral/medial slope ratio. MRI, magnetic resonance imaging.

Seven studies evaluated tibial slope as a risk factor. Four studies measured both the MTS and LTS using Hudek's technique [33] on MRI [13, 22, 26, 38], while one study [51] measured the tibial slope on MRI using Hashemi's technique. Three other studies measured the MTS on knee radiographs [43, 50, 59] (Figure 5).

#### MTS on MRI

**Figure 6:**
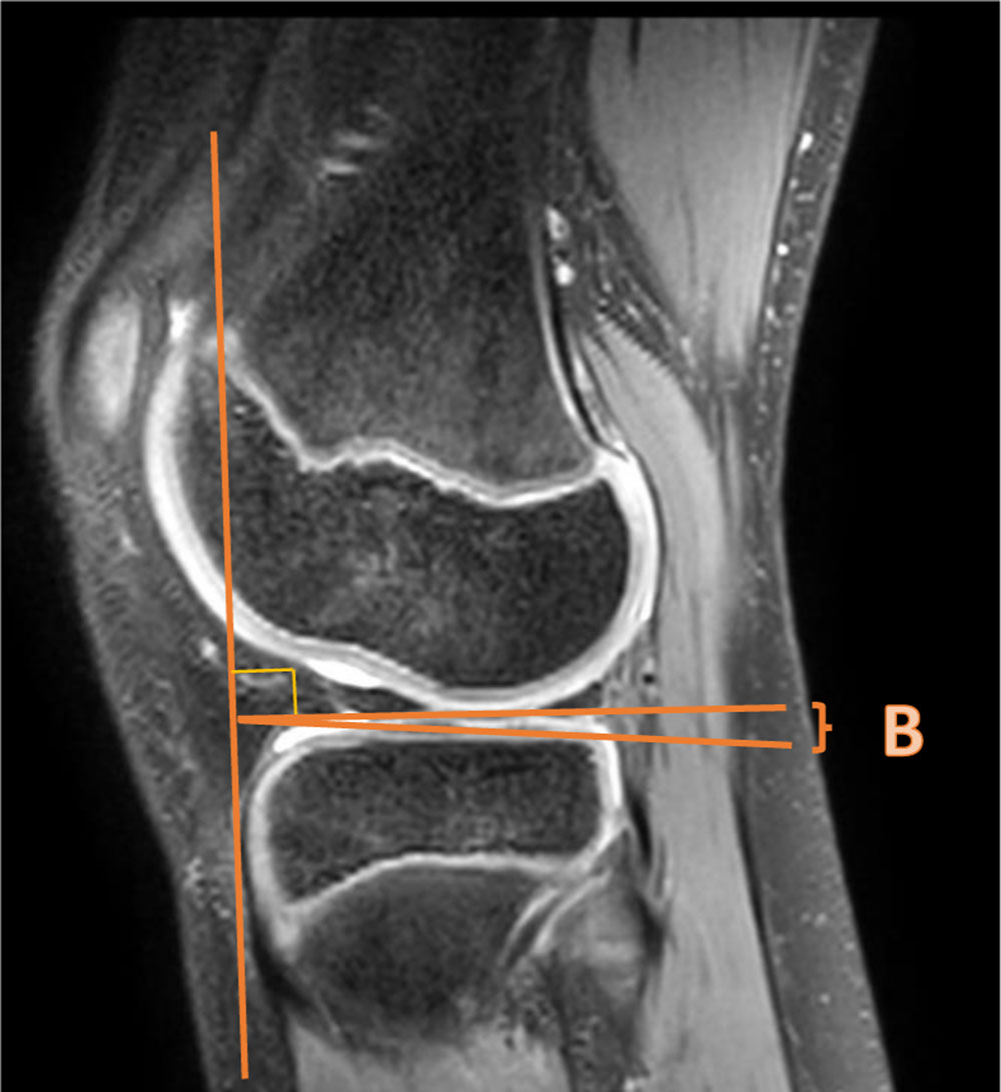
Lateral tibial slope. Lateral tibial slope was measured by Hudek's technique [33].

According to the meta‐analysis, the average MTS (Figure 4) was significantly higher in the ACL‐injured group compared to the control group, with a mean difference of 0.55° (95% CI: 0.09–1.01, *p* = 0.02), fixed effect model (Figure 6a). The average MTS was in the ACL‐injured group 4.8° versus 3.6° in the control group. This is in contrast with the study of Shaw et al. [51], who found no significant difference in tibial slope between the ACL‐injured group and the control group. Notably, this latter study measured the MTS on MRI using Hashemi's technique [29].

#### MTS on knee radiographs

The MTS on knee radiographs was measured in three studies [43, 50, 59] with the same measuring technique [29]. These studies were included in the meta‐analysis. On average, the posterior tibial slope of ACL‐injured patients was higher compared to the control group, 10.4° versus 8.6°, respectively, with a mean difference of 1.60° [95% CI: 0.80–2.39, *p* < 0.0001], fixed effect model (Figure 6b).

#### LTS on MRI

According to the meta‐analysis, the LTS (Figure 6) of the ACL‐injured group was also, on average, higher compared to the control group, with a mean difference of 2.04° (95% CI: 0.75–3.32, *p* = 0.0003), random effect model (Figure 6c). The average LTS was in the ACL‐injured group 4.3° versus 2.8° in the control group. This is in contrast with the study of Shaw et al. [51], which measured the lateral tibial slop on MRI using Hashemi's technique [29] and found no significant difference in the tibial slope between the ACL‐injured group and the control group.

#### Logistic regression on LTS

Edwards et al. performed logistic regression analyses. They found that the LTS is a significant predictor for ACL injury (odds ratio [OR]: 1.58, 95% CI: 1.18–2.13, *p* = 0.002) [22]. The MTS was not identified as a significant predictor (OR: 1.05, 95% CI: 0.83–1.32, *p* = 0.687).

#### LTS/MTS ratio on MRI

Two studies measured the LTS/MTS ratio on MRI and were included in a meta‐analysis [13, 22]. The LTS/MTS ratio was in the ACL‐injured group on average higher compared to the control group, with a mean difference of 0.54° (95% CI: 0.30–0.78, *p* < 0.0001), fixed effect model (Figure 6d).

#### Medial and lateral slope depth on MRI

Shaw et al. [51] utilized sagittal plane images to measure both the MTS and LTS depth. The study found no difference in LTS depth between the controls and the ACL‐injured group, but the study did find a significant difference in MTS depth (*p* = 0.01).

### Patellar height

**Figure 7:**
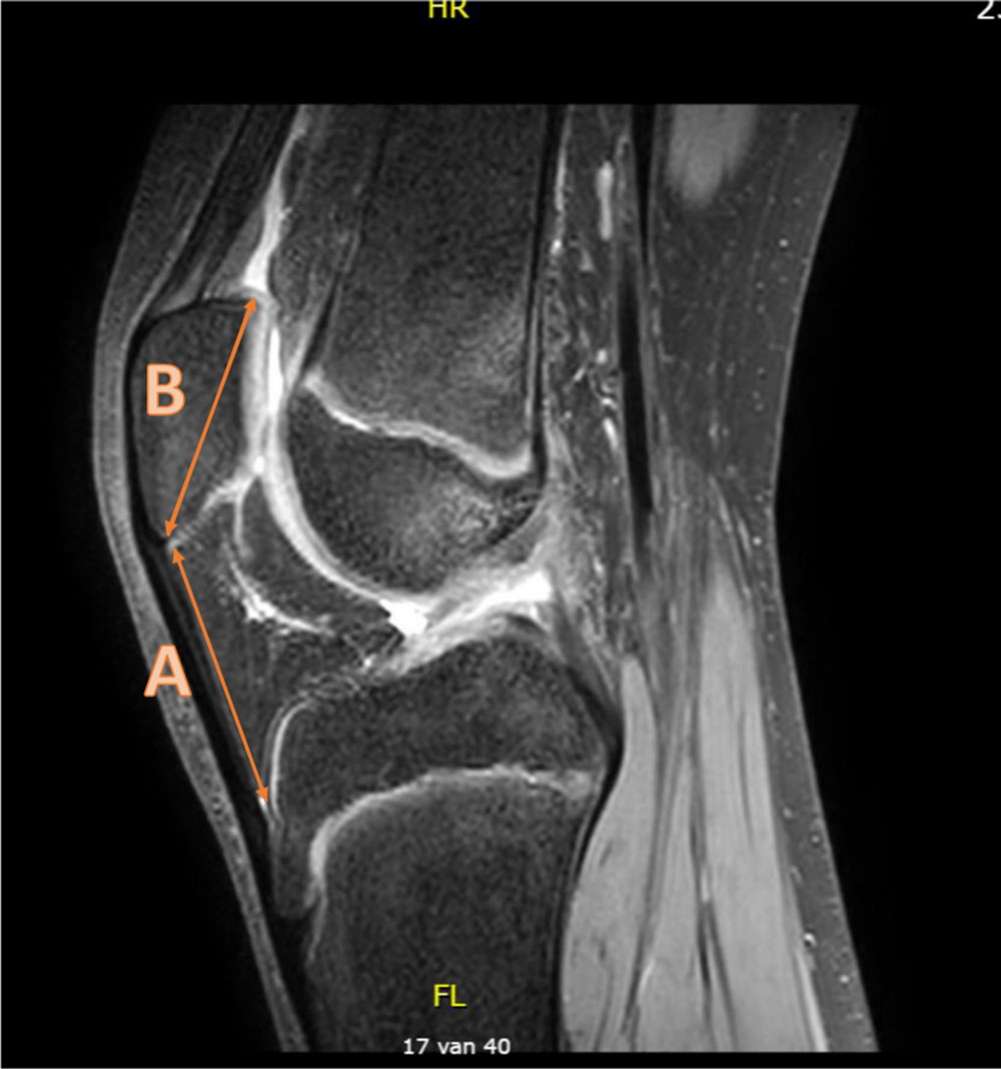
Patellar height measured by Insall–Salvati. This graphical representation shows patellar tendon length measurements (A) and patella length (B) based on MRI examination of knee. Insall–Salvati ratio is calculated from the ratio of A/B [34]. MRI, magnetic resonance imaging.

**Figure 8:**
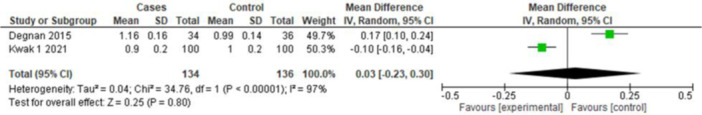
Meta‐analysis of patellar height. CI, confidence interval; SD, standard deviation.

Two studies measured the influence of patellar height measured by the Insall–Salvati technique on MRI (Figure 7) on ACL injuries [14, 37]. The meta‐analysis showed that patellar height was not significantly different in the ACL‐injured group compared to the control group, with a mean difference of 0.03 (95% CI: −0.23 to 0.30, *p* = 0.80), random effect model (Figure 8). The average Insall–Salvati ratio of both groups was 1.0.

## DESCRIPTIVE OUTCOMES OF MORPHOLOGICAL RISK FACTORS

### Femoral morphological risk factors

#### Intercondylar spine

Freychet et al. assessed the presence of an intercondylar spine on the MRI. This is described as a spine opposite to the anterior part of the femoral notch [27]. The intercondylar spine was significantly more often present in the ACL‐injured group compared to the control group in 18 of the 49 cases versus 7 of the 40 cases (*p* < 0.02), respectively.

#### Femoral trochlea dysplasia

Kwak et al. assessed femoral trochlea dysplasia in both ACL‐injured and control groups [37]. The diagnosis of dysplasia type was based on the Dejour classification [36], which stratifies dysplasia as Types A through D. Type A dysplasia is characterized by a shallow trochlea (>145°), Type B involves a flat trochlea, Type C encompasses medial hypoplasia and Type D includes a cliff. In the ACL‐injured group, 56 (48.3%) knees exhibited trochlear dysplasia, including 51 (91.1%) Dejour Type A; and in the control group, 12 (10.4%) knees exhibited trochlear dysplasia, 12 (100%) Dejour Type A. The prevalence of femoral trochlear dysplasia was significantly higher in the ACL‐injured group than in the control group (*p* < 0.001).

#### Femoral condyle

Akcaalan et al. [1] performed MRI in children, aged 5–11 years, in ACL‐injured patients and healthy ACL patients. Several measurements were performed regarding the femoral condyle, such as the medial femoral condyle height, the transcondylar width and the intercondylar height. The medial femoral condyle height and transcondylar width were significantly (*p* < 0.001) bigger in the ACL‐injured group compared to the control group. The intercondylar width was significantly (*p* = 0.028) smaller in the ACL‐injured group compared to the ACL‐injured group.

#### Anatomic differences in tibia and femur

Kwak et al. [38] performed MRI in both ACL‐injured patients and a control group. The study assessed whether there were anatomic differences in the tibia and the femur. The anterior‐posterior length as well as the sagittal curvature was assessed in millimetres for both the lateral/medial femur and lateral/medial tibia. The sagittal curvature of the medial tibia was significantly shorter in the ACL‐injured group versus the control group, 44.9 ± 10.4 versus 57.4 ± 10.7 (*p* < 0.01), respectively. The sagittal curvature of the lateral tibia was also significantly shorter in the ACL‐injured group versus the control group, 39.6 ± 8.4 mm versus 43.1 ± 8.5 mm (*p* < 0.01), respectively. The lateral femoral sagittal curvature was significantly longer in the ACL‐injured patients versus the control group, 23.4 ± 3.5 mm versus 22.0 ± 3.5 mm (*p* < 0.01), respectively. No significant difference was found in the anteroposterior length of both the femur and tibia.

### Other morphological risk factors

#### Meniscal bone angle

Edwards et al. measured the lateral meniscal bone angle (LMBA), as described by Sturnick et al. [56]. In this technique, the tangent is drawn across the superior meniscal surface of the posterior horn of the lateral meniscus, and the acute angle between this line and the tangent to subchondral bone of the tibial plateau. Edwards found no significant difference between the ACL‐injured group and the control group [22]. Edwards also assessed the LTS/LMBA ratio of both groups and found this to be a risk factor with significance between the groups (*p* = 0.0001) [22]. This ratio was found to be the strongest predictor of ACL injury in this study, with an OR of 3.13 (95% CI: 1.48–6.62, *p* = 0.003).

#### Meniscal slope

Freitas et al. assessed the meniscal slope. This study found that the medial meniscal slope was significantly higher in the ACL‐injured group, 1.8° versus 0.1° (*p* < 0.001) [26]. The lateral meniscal angle was significantly higher in the ACL‐injured group, 3.2° versus −1.2° (*p* < 0.001) [26].

#### Roof inclination angle

Samora et al. measured the roof inclination angle (RIA) [50]. The RIA was significantly lower in ACL‐injured patients compared to the control group, 38.1° versus 40.3° (*p* = 0.001).

#### Tibial eminences

The study of Shaw et al. assessed the tibial eminence (TE) as a risk factor for ACL injury in skeletally immatures [51]. In this study, the height, volume, and width of the TE were determined on MRI using Hashemi's technique. Neither height, width or volume was significantly different in ACL‐injured patients compared to the control group. This is in contrast to the study performed by Akcaalan et al. [1], where the tibial spine height was significant (*p* < 0.001) higher in children (aged 5–11 years), yet no significant (*p* = 0.736) difference in tibial spine height was found in adolescents (12–19 years) between ACL‐injured patients and control group.

## DISCUSSION

The main finding of the present systematic review is that a smaller NWI, steeper MTS and LTS and higher LTS/MTS ratio are relevant knee morphological factors for ACL injury in skeletally immature patients. The majority of these parameters were determined on MRI. These findings confirm the hypothesis that the tibial slope and the notch width index play an important role morphological role in ACL injuries in skeletally immature patients.

In 1938, Palmer et al. were the first to recognize the role of a small intercondylar notch in sustaining an ACL injury in adults [45]. Zeng et al. confirmed through a meta‐analysis that a lower NWI, both measured on MRI and x‐ray, predisposes adults to ACL injury [67]. Theoretically this is related to a small notch that may constitute a mechanical impingement on the ACL during internal rotation and hyperextension of the knee [65].

In skeletally immature patients, the diagnostic methodology may impact the identification of potential morphological risk factors. In this systematic review, the NWI measured on MRI was a relevant factor for ACL injury in skeletally immature patients. Yet interestingly, no difference in NWI between the skeletally immature ACL‐injured group and the control groups was measured on knee radiographs [50, 59]. This might be explained by the fact that the accuracy of measurements on radiographs might be influenced by slight variations in rotation and angulation, the reason for which Herzog et al. recommended the use of MRI for better reliability and accuracy of NWI measurement in adults [30]. This later study has not been conducted in skeletally immature patients; however, it can also be assumed that small variations in rotation and angulation may influence the measurements in this population.

The NWI also changes with the age of the patient and decreases from childhood to adolescence [21]. This must be taken into consideration when interpreting the results from this systematic review, where patients with open physes were included with ages varying from 7 to 16 years. In the present study the difference between NWI in ACL‐injured patients and control patients was only small, respectively 0.25 and 0.26, in adults it has been shown that patients with an NWI > 0.25 were at risk for ACL injuries [32]. The study of Hoteya et al. demonstrates that the cutoff point of 0.25 NWI is significant in adults, indicating that even small variations can substantially increase the risk of ACL injuries.

In the present review, both the MTS and LTS were identified as relevant knee morphological factors for ACL injuries in skeletally immature patients on MRI. This is in line with the finding of Wang et al., who found that these two morphological factors were both significant overall risk factors in adults [60].

This may be explained from a biomechanical perspective, where the force generation on the ACL is greatest when the tibia is anteriorly translated in valgus and internally rotated [10]. In adults, a significant increase in tibial rotation and anterior translation has been observed with a steeper tibial slope [40]. It has been suggested that the steeper tibial slope is a major risk factor for a significant increase in ACL force [7, 16]. These forces are accentuated in skeletally immature athletes, who experience frequent axial loading during sports [3]. The steepness of the tibial slope also plays a role in re‐injury rates in this population. Dietvorst et al. asserted that children with an LTS exceeding 7° are at a significantly elevated risk of re‐injury [18]. In the present included studies, most tibial slopes were measured using MRI. However, in the current literature, tibial slope is most commonly measured on plain lateral knee radiographs [61]. In the present study, the mean difference for the MTS, as measured on MRI, was minimal. However, one cannot assume that differences in tibial slope observed on MRI are equivalent to those measured on knee radiographs and caution should be used interpretating this present study with the different modalities used [63].

Both O'Malley et al. [43] and Vyas et al. [59] found that the MTS on radiographs was not increased in ACL injuries in skeletally immature patients. One of the concerns of using radiographs is that the LTS and MTS are superimposed, which might result in ignoring the asymmetry of the medial and lateral aspects of the tibia. This is relevant in skeletally immature patients since the articular cartilage represents the function point of the tibiofemoral articulation, which is not visible on radiographs [8]. Minor rotational changes of the tibia on the lateral radiograph influence the measured tibial slope as well [24]. In adults, the mean slope increase was 3° at 40° of tibial rotation [58]. Although this later study was conducted in children, it can be inferred that knee rotation may lead to discrepancies between the measured tibial slope and the actual anatomical slope. MRI seems to be the best modality for measuring tibial slope in skeletally immature patients, mentioning that the measurements from radiographs and MRI scans cannot be used interchangeably, and caution should be used when interpreting and comparing studies using measurements of the tibial slope [35, 48]. In the present study, the average tibial slope measured on plain knee radiographs in the ACL‐injured group was 10.4°, and the control group was 8.6°. A recent study in adults has demonstrated that a tibial slope >10° is a risk factor for sustaining an ACL injury, confirming the current study in skeletally immature patients where the average tibial slope was 10.6° in ACL injured [23]. In adults, a minimally clinically significant difference of 2° in tibial slope, measured on plain lateral knee radiograph, has been defined [11, 28, 49]. In the present study, the mean difference was 1.6°, which is smaller than the minimally clinically significant difference established in adults. However, it remains uncertain whether this threshold for tibial slope is applicable to skeletally immature patients. To date, no studies have specifically addressed this question.

Recent increases in ACL injuries in children and adolescents have led to more interest in age‐specific surgical treatment options [4]. However, the current operative treatment comes with a relatively high percentage of re‐rupture rates in skeletally immature patients compared to adults [15, 62]. Knee morphology might play a role in this high re‐rupture rate [18]. During childhood and adolescence, many anatomical features may undergo more complex changes than simply scaling with body size. Given that these changes may impact ACL function, consideration of growth and maturation may be key when considering the treatment of paediatric ACL injuries [12]. NWI decreases from childhood to adolescence in skeletally immature patients without ACL injury [21]. Engebretsen et al. described that the ACL injury itself also alters the growth of the knee. They found that the lateral posterior tibial slope increased more in the ACL‐injured knee than in the contralateral uninjured knee in a group of skeletally immature patients [41].

The assessment of potential morphological risk factors as identified in the current review may have clinical implications in the treatment of ACL injuries in children and adolescents. Some of the described morphological factors associated with ACL injury might be modifiable via surgery. In adults, anterior closing‐wedge osteotomy should be considered in patients with a tibial slope of ≥12° who have a failure of ACL reconstruction [5, 16, 54]. Slope corrections in skeletally immature patients by temporary epiphysiodesis or osteotomies in adolescents with deviant tibial slopes could be considered.

The present systematic review and meta‐analyses have several limitations. The level of evidence for the included studies is relatively low, as nearly all of them were case‐control studies. The heterogeneity of applied imaging techniques and methods to measure potential risk factors complicated the pooling and interpretation of data. The majority of parameters were only described as differences between patient and control groups. Associations between morphology and ACL injury were rarely provided by pathologic value or an associated odds ratio. Furthermore, not all known knee morphological factors from studies in adults have been investigated yet in skeletally immatures [15]. As such, no statements can be made about potential other relevant factors such as Q‐angle, ACL morphology, and cartilage morphology. Another limitation is that most of the studies included in the meta‐analyses were performed in a retrospective design. These retrospective case‐control studies are more susceptible to selection bias.

Future studies should assess whether the identified morphological parameters are truly risk factors for primary ACL injury and possibly also risk factors for re‐ruptures in this population. In the present search, no articles were found assessing the sagittal alignment factors of the knee in paediatric ACL injuries. Future studies should explore the influence of these factors on paediatric ACL injuries. Studies should also assess and validate the technique and optimal imaging modality for these morphological factors. Future research should assess how and if these morphological factors in skeletally immatures develop during growth.

## CONCLUSION

A small notch width index, steeper MTS and LTS, and a higher LTS/MTS ratio were identified as relevant knee morphological factors for ACL injuries in skeletally immature patients. There may still be other potential risk factors, but research on these aspects remains insufficient. Future studies should identify the influence of growth on these factors and assess if these are risk factors for graft failures after ACL reconstruction.

## AUTHOR CONTRIBUTIONS

**Robin Voskuilen**: conception and design of the study, data extraction and selection, analyses and interpretation, drafting the manuscript. **Martijn Dietvorst**: conception and design of the study, data extraction and selection, interpretation of the data and drafting the manuscript. **Marieke van der Steen**: conception and design of the study, data analysis and interpretation, revision of the manuscript. **Rob P. A. Janssen**: conception and design of the study, data interpretation and revision of the manuscript. All authors read and approved the final manuscript.

## CONFLICT OF INTEREST STATEMENT

The authors declare no conflicts of interest.

## ETHICS STATEMENT

The ethics statement is not available.

## Data Availability

Research data are not shared.
